# A Treatise on the Use of the Sympathetic Nerve and Its Ganglions, with Their Influence on Various Diseases of the Anatomical and Pelvic Viscera

**Published:** 1844-10

**Authors:** 


Art. III.—*A Treatise on the Use of the Sympathetic Nerve and its Ganglions, with their Influence on various Diseases of the Abdominal and Pelvic Viscera.* By T. B. Procter, m.d. m.r.c.s., &c. With Three Lithographic Plates.—*London,* 1844. 4to, pp. 48.

Our want of precise knowledge in regard to the functions of the sympathetic nerve must be freely admitted ; and any contribution to an improved acquaintance with its structure and offices must be gladly hailed by the anatomist and physiologist. Our first glance at this work led us to hope that it might contain some really original facts or deductions; but, we regret to say, that a further examination of it has left our knowledge of the subject very much where it found it. Dr. Procter has added no new fact to the anatomy of the sympathetic nerve; and in regard to its physiology, his dominant idea appears to be, that it exerts a regulative power over the supply of blood to particular organs and its flow through them, (and consequently over the processes of nutrition, secretion, &c.) by its action upon the contractile coats of the arteries. In support of this view, he only adduces a single experiment of his own, and seems ignorant of much that has been done by others. The doctrine in question has been already very clearly stated by Dr. Carpenter in his 'Human Physiology,' (§§ 423, 502, 504;) and much more evidence is there adduced in its favour, within the compass of a few lines, than is contained in Dr. Procter's quarto treatise. His single experiment, however, is sufficiently interesting to deserve mention. "A horse was killed by dividing the medulla in the French way, the bowels turned aside, and the branch of the sympathetic nerve, which joins the ischiatic, laid bare; also one of the arteries of the leg. A wire, applied to the positive pole of a galvanic battery, defended with sponge, was applied to the nerve; and the negative wire to the artery : the positive wire was then drawn slowly along the plates of a fifty-plate battery, and the effect was certainly not only to reproduce the pulsation in the artery, but also clearly to excite circulation in the more minute vessels." As a proof that he was not unduly influenced by his preformed theory, in relating the resuts of his experiment, (which he has subsequently repeated with the same result,) Dr. Procter mentions that one of the knacker's men said, "see how that *pipe* beats when they put on those wires;" and that the rest of the men expressed themselves satisfied of the fact.

Dr. Procter adds, in an appendix, some cases in which strychnia was given, to excite what he deemed the indolent action of the sympathetic nerve in amenorrhea, impotence, &c.; and with successful results. He does not, however, mention the doses in which it was given, nor does he give any *unsuccessful* cases. We recommend him, therefore, to give a fuller account of his practice, for the benefit of his professional brethren.

